# Uptake and correlates of chlamydia and gonorrhea testing among female sex workers in Southern China: a cross-sectional study

**DOI:** 10.1186/s12889-021-11526-w

**Published:** 2021-07-28

**Authors:** Pei Zhen Zhao, Ya Jie Wang, Huan Huan Cheng, Ye Zhang, Wei Ming Tang, Fan Yang, Wei Zhang, Ji Yuan Zhou, Cheng Wang

**Affiliations:** 1 State Key Laboratory of Organ Failure Research, Ministry of Education, and Guangdong Provincial Key Laboratory of Tropical Disease Research, Department of Biostatistics, School of Public Health, Southern Medical University, Guangzhou, China; 2 Dermatology Hospital, Southern Medical University, Guangzhou, China; 3 Southern Medical University Institute for Global Health and Sexually Transmitted Diseases, Guangzhou, Guangdong China; 4 The Third Affiliated Hospital, Sun Yat-Sen University, Guangzhou, China; 5 Kirby Institute, New South Wales University, Sydney, Australia

**Keywords:** Female sex workers, Chlamydia testing, Gonorrhea testing

## Abstract

**Background:**

Female sex workers (FSW) are highly susceptible to chlamydia and gonorrhea infection. However, there is limited literature examining their testing uptake to date. This study aimed to assess the uptake and determinants of chlamydia and gonorrhea testing among FSW in Southern China.

**Methods:**

A cross-sectional study with convenience sampling was performed in five cities in Southern China. Data on socio-demographic characteristics, sexual behaviors, chlamydia and gonorrhea testing, and the utilization of health care services from participants were collected through face-to-face interviews. Univariate and multivariable logistic regressions were used to determine factors associated with chlamydia and gonorrhea testing, respectively.

**Results:**

Overall, 1207 FSWs were recruited, with the mean age of 30.7 ± 6.8 years and an average number of clients of 7.0 (4.0–10.0) per week. 65.4% participants constantly used condoms with clients during the past month. Only 7.5 and 10.4% had been tested for chlamydia and gonorrhea in the last year, respectively. Multivariable analysis indicated that FSW who worked at low tiers (adjusted Odds Ratio (a*OR*) = 2.36, 95%CI:1.23–10.14), had more clients in the last month (a*OR* = 1.03, 95%CI:1.01–1.05), used condoms consistently (a*OR* = 1.79, 95%CI:1.12–2.86), had STD symptoms (a*OR* = 4.09,95%CI:2.62–6.40), had been tested for HIV (a*OR* = 5.16, 95%CI:3.21–8.30) or syphilis (a*OR* = 6.90, 95%CI:4.21–11.22) in the last year were more likely to have chlamydia testing. In addition, FSW who had more clients in the past month (a*OR* = 1.02,95%CI:1.00–1.04), had STD symptoms (a*OR* = 3.33, 95%CI:2.03–5.46), had been tested for HIV (a*OR* = 3.94, 95%CI:2.34–6.65) and syphilis (a*OR* = 3.27, 95%CI:1.96–5.46) in the last year were more likely to have gonorrhea testing.

**Conclusions:**

The testing rates of chlamydia and gonorrhea are low among Chinese FSW. Integrating chlamydia and gonorrhea testing into HIV testing promotion programs may help bridge the gap among FSW.

**Supplementary Information:**

The online version contains supplementary material available at 10.1186/s12889-021-11526-w.

## Background

Chlamydia and gonorrhea are two major causes of reproductive tract morbidity [1], which could increase the risk of both acquiring and transmitting of human immunodeficiency virus (HIV) [2]. FSW are incredibly vulnerable to chlamydia and gonorrhea due to their frequent sexual contact with numerous concurrent sexual partners [3]. In China, the prevalence of chlamydia among FSW is 13.6%, while that of gonorrhea is 6.1% [4], much higher than that of general 15–49 years old women (chlamydia 3.8%, and gonorrhea 0.9%) [5]. This situation is much worse among FSW from middle and low tier (8.0 and 20.0%, respectively) [6]. Middle-tier venues referred to hair salons, barber shops, massage parlors, roadside shops, foot-bathing shops, guesthouses and roadside restaurants, while low-tier venues included streets and other public outdoor places [7].

Untimely diagnosis and treatment of chlamydia and gonorrhea can lead to onward transmission [8] and serious complications, including pelvic inflammatory disease (PID), miscarriage, infertility, premature delivery, ectopic pregnancy and low birthweight [9]. Testing is a cost-effective strategy to increase the awareness of being infected or not, and thus reduce the potential onward transmission of chlamydia and gonorrhea among FSW [10]. However, the current sexually transmitted disease (STD) prevention services in the low- and middle- income countries (LMIC) mainly focused on controlling HIV and syphilis [11],including China, while to date, limited studies are examining the chlamydia and gonorrhea testing uptake among FSW [7, 12]. Previous studies in the United States and Vietnam indicated that the asymptomatic infection of chlamydia and gonorrhea [13], low levels of knowledge [14], misperceptions of risk, stigma around STD [15], and cost [16] are the main barriers for improving testing uptake of chlamydia and gonorrhea among women who are actively engaged in sex. However, the uptake of chlamydia and gonorrhea testing and their influencing factors among FSW still remain unclear in China.

This study aimed to investigate chlamydia and gonorrhea testing rate and associated factors among FSW in China.

## Methods

### Study sites

We conducted a venue-based cross-sectional study in Guangdong Province in Southern China. Guangdong Province was chosen for this study because of a high burden of chlamydia and gonorrhea. In 2018, the average burden of chlamydia and gonorrhea in China was 50.3 and 9.6 cases per 100,000 population, whereas it was reported as 70.1 and 27.9 cases per 100,000, respectively in Guangdong Province [17]. Guangdong province has consistently ranked first by the number of newly reported chlamydia and gonorrhea cases in the last decade in China [18].

### Study participants

Five cities (Jiangmen, Zhuhai, Yunfu, Jieyang and Yangjiang) in Guangdong Province were selected to implement this research between August 2018 and December 2018 based on local capacity of FSW outreach programs, and to encompass areas where STD were highly prevalent.

Each of the five cities had a FSW outreach team, and each team had at least one public health staff and one medical staff (nurse or physician). These teams had extensive FSW outreach services experience, which were able to provide regular services including public health interventions such as condom promotion, reproductive health and STD counseling [19]. Participants were eligible for participation if they met the following inclusion criteria:1) born biologically as a female; 2) aged 18 years or above; 3) exchanged sex at least once for goods or money during the past year; 4) willing to participate and complete the survey by providing written informed consent. The women were also eligible if they only had sex with a few boyfriends consequently in the past year who did provide housing for them.

### Sample size

The primary outcome of this study was the testing rates of chlamydia and gonorrhea in the past 12 months. A previous study reported a detection rate of 65.4% for chlamydia and 77.1% for gonorrhea among FSW [12]. We applied Two-sided Confidence Intervals (CI) for One Proportion method to estimate a sample size of 957 for this study to produce a two-sided 95% CI and a width of 0.060. According to the number of FSW at each city, the total number of study participants at each city ranged from 200 to 300.

### Data collection

Paper questionnaires were used for data collection in research. The questionnaire items in this study were created based on discussions with HIV and STD experts, local FSW outreach service staff, and policy makers (Additional file 1). We also piloted the survey with 20 volunteer female sex workers to test questionnaire items and examine local FSW outreach service capacity. The pilot data were not included in the final analysis.

Prior to this study, a mapping of the sex work venues was performed by local outreach team in each study site according to geographic area and type of venue. Convenience sampling was used to enroll FSW from selected middle- and low- level venues. The information was collected in FSW’s working venues and only one person from the outreach team appeared during each face-to-face interview. Each paper questionnaire was completed by eligible participants themselves with the help of FSW outreach workers. For those who were not able to read the questionnaire, the outreach workers would help to fill out the survey by asking them questions of the survey. All survey data were anonymous and confidential, and written consent was obtained prior to the beginning of the survey. Each participant was given 30 Yuan (about 4.3 USD) for incentive of participation.

### Measures

#### Social-demographic and behavioral variables

Socio-demographic information included age, ethnicity, marital status, household registration, working duration in the investigated city, highest education level obtained and annual income. Sexual behavioral variables included average number of clients, condom use, anal sex, and STD symptoms (abnormal vaginal discharge, vaginal bleeding after sexual contact, urethral discharge, dysuria, and vaginal pruritus). Inconsistent condom use was defined as not always using condoms when engaged in commercial sex.

#### STD testing variables

Information on testing for HIV, syphilis, chlamydia and gonorrhea (all in binary) in the past year was obtained in this study. The primary outcome measure was whether or not having chlamydia or gonorrhea testing (0 = non-testing, 1 = testing) over the past 12 months.

### Statistical analysis

The study data were double-entered into the Epidata 3.0 software (Epidata Association from Denmark). Descriptive analysis was used to describe demographic factors, sexual behaviors and STD testing. Categorical data were presented as the number and percentage of FSW. Continuous data were expressed as $\overline{x}\pm SD$ if data were normally distributed, and as *median* (*P*_25_ ~ *P*_75_) if the data did not present normal distribution. The Chi-square test was used to compare categorical variables between groups.

Univariate and multivariable logistic regressions were conducted to determine variables related to chlamydia and gonorrhea testing in the past 12 months. In the multivariable model, we adjusted for age, ethnicity, marital status, the highest education level, monthly income, household registration and length of time working in the local city. We reported odds ratios (*OR*), 95% confidence intervals (*CI*) and *P* values. Results are deemed to be statistically significant when *P* ≤ 0.05. All analyses were conducted on SAS (version 9.4, SAS int. Cary, NC, USA).

## Results

### Sociodemographic characteristics

Overall, 1261 women were recruited in this survey. Fifty-four individuals declined to participant the study. Finally, a total of 1207 participants completed the survey. The mean age was 30.7 ± 6.8 years old. The majority of the participants were between 25 and 30 years old (30.6%, 369/1207), married (63.1%, 762/1207), had a monthly income between $500–$1000 (56.8%, 686/1207) and worked in the current location over 1 year (61.1%,738/1207). Almost three-fourths (77.6%, 936/1207) of participants’ household registrations were in other provinces (Table 1).

**Table 1:** Social demographic and sexual behavioral characteristics of participants among FSW in Southern China, 2018 (*N* = 1207)

Characteristics	Gonorrhea testing in the past 12 months ***n*** (%)			Chlamydia testing in the past 12 months ***n*** (%)			Total
	No (***n*** = 1117)	Yes (***n*** = 90)	***P***	No (***n*** = 1082)	Yes (***n*** = 125)	***P***	
**Age (years)**			< 0.001			< 0.001	
18–25	182(16.3)	19(21.1)		177(16.4)	24(19.2)		201(16.7)
26–29	356(31.9)	13(14.4)		345(31.9)	24(19.2)		369(30.6)
30–34	299(26.8)	15(16.7)		292(27)	22(17.6)		314(26.0)
≥ 35	280(25.1)	43(47.8)		268(24.8)	55(44.0)		323(26.7)
**Workplace**			0.040			0.003	
Middle tier	128(11.5)	4(4.4)		128(11.8)	4(3.2)		132(10.9)
Low tier	989(88.5)	86(95.6)		954(88.2)	121(96.8)		1075(89.1)
**Ethnicity**			0.002			0.004	
Han	1011(90.5)	72(80.0)		980(90.6)	103(82.4)		1083(89.7)
Non-Han	106(9.5)	18(20.0)		102(9.4)	22(17.6)		124(10.3)
**Marital status**			< 0.001			0.004	
Not married	315(28.2)	26(28.9)		303(28)	38(30.4)		341(28.3)
Married	717(64.2)	45(50.0)		700(64.7)	62(49.6)		762(63.2)
Divorced or widowed	84(7.5)	19(21.1)		78(7.2)	25(20.0)		103(8.5)
**Household registration**			0.071			< 0.001	
Local city	75(6.7)	11(12.2)		67(6.2)	19(15.2)		86(7.1)
Other cities in this province	168(15.0)	17(18.9)		153(14.1)	32(25.6)		185(15.3)
Other provinces	874(78.2)	62(68.9)		862(79.7)	74(59.2)		936(77.6)
**Monthly income (USD)**			0.017			0.001	
< $500	260(23.3)	33(36.7)		246(22.7)	47(37.6)		293(24.3)
$500–$1000	644(57.7)	42(46.7)		627(57.9)	59(47.2)		686(56.8)
> $1000	213(19.1)	15(16.7)		209(19.3)	19(15.2)		228(18.9)
**Education level**			0.669			0.272	
Illiterate or elementary school	287(25.7)	27(30.0)		284(26.2)	30(24.0)		314(26.0)
Junior high school	737(66)	56(62.2)		713(65.9)	80(64.0)		793(65.7)
High school and above	93(8.3)	7(7.8)		85(7.9)	15(12.0)		100(8.3)
**Length of time working in current location**			< 0.001			< 0.001	
1 year and above	665(59.5)	73(81.1)		634(58.6)	104(83.2)		738(61.1)
6–12 months	267(23.9)	15(16.7)		264(24.4)	18(14.4)		282(23.4)
< 6 months	184(16.5)	2(2.2)		183(16.9)	3(2.4)		186(15.4)
**Average number of clients in the past month**			< 0.001			< 0.001	
< =30	626(56.0)	32(35.5)		612(56.5)	46(36.8)		658(54.5)
31–60	352(31.5)	51(56.7)		333(30.8)	70(56.0)		403(33.4)
> 60	139(12.4)	7(7.8)		137(12.7)	9(7.2)		146(12.1)
**Consistent condom uses with clients in the past month**			0.969			0.099	
No	387(34.6)	31(34.4)		383(35.4)	35(28.0)		418(34.6)
Yes	730(65.4)	59(65.6)		699(64.6)	90(72.0)		789(65.4)
**Providing anal sex for clients**			< 0.001			< 0.001	
No	903(80.8)	57(63.3)		889(82.2)	71(56.8)		960(79.5)
Yes	214(19.2)	33(36.7)		193(17.8)	54(43.2)		247(20.5)
**Regular partner (boyfriend or husband)**			0.153			0.015	
No	273(24.4)	16(17.8)		270(25)	19(15.2)		289(24.0)
Yes	843(75.5)	74(82.2)		811(75)	106(84.8)		917(76.0)
**HIV testing in the past 12 months**			< 0.001			< 0.001	
No	782(70.0)	31(34.4)		773(71.4)	40(32.0)		813(67.4)
Yes	335(30.0)	59(65.6)		309(28.6)	85(68.0)		394(32.6)
**Syphilis testing in the past 12 months**			< 0.001			< 0.001	
No	778(69.7)	33(36.7)		777(71.8)	34(27.2)		811(67.2)
Yes	339(30.3)	57(63.3)		305(28.2)	91(72.8)		396(32.8)
**STD symptoms in the past 12 months**			< 0.001			< 0.001	
No	665(59.5)	11(12.2)		659(60.9)	17(13.6)		676(56.0)
Yes	452(40.5)	79(87.8)		423(39.1)	108(86.4)		531(44.0)
**Smoke in the past 12 months**			0.646			0.049	
No	909(81.4)	75(83.3)		874(80.8)	110(88.0)		984(81.5)
Yes	208(18.6)	15(16.7)		208(19.2)	15(12.0)		223(18.5)
**Sexual abuse in the past 12 months**			< 0.001			< 0.001	
No	1030(92.2)	69(76.7)		1018(94.1)	81(64.8)		1099(91.1)
Yes	87(7.8)	21(23.3)		64(5.9)	44(35.2)		108(8.9)

### Sexual behaviors

A median number of 28.0 (16.0–40.0) clients was reported over the past month. Most participants used condoms consistently when engaging in sex with clients in the past month (65.4%, 789/1207) and had a regular partner (76.0%, 917/1207). One-fifth (20.5%, 247/1207) of the participants had ever provided anal sex for clients. Approximately half (44.0%, 531/1207) of the participants had STD-related symptoms over the past 12 months (Table 1).

### Chlamydia and gonorrhea testing

The testing rate of gonorrhea and chlamydia in the past 12 months were 7.5% (90/1207) and 10.4% (125/1207). Seventy-five (6.2%, 75/1207) participants had both gonorrhea and chlamydia testing over the past year. Additionally, the testing rate of HIV and syphilis were 32.6% (394/1207) and 32.8% (396/1207) in the past year.

### Factors correlated with chlamydia testing

In the multivariable logistic analysis, chlamydia testing was positively correlated with low tier (adjusted OR (a*OR*) = 3.53, 95%CI:1.23–10.14), number of clients in the past month (a*OR* = 1.03, 95%CI:1.01–1.05), consistent condom use with clients in the past month (a*OR* = 1.79 95%CI:1.12–2.86), providing anal sex for clients (a*OR* = 2.36, 95%CI:1.54–3.60), having regular partners (a*OR* = 3.12, 95%CI:1.72–5.68), having STD symptoms in the past 12 months (a*OR* = 4.09, 95%CI:2.62–6.40), having HIV testing in the past 12 months (a*OR* = 5.16, 95%CI:3.21–8.30), having syphilis testing in the past 12 months (a*OR* = 6.90, 95%CI:4.21–11.22) and sexual abuse in the past 12 months (a*OR* = 4.42, 95%CI:2.64–7.39) after adjusting for age, ethnicity, marital status, the highest education level, marital status, monthly income, household registration, and length of time working in the current location Table 2).

**Table 2:** Factors associated with chlamydia testing among FSW in Southern China, 2018 (*N* = 1207)

Characteristics	Crude Model		Adjusted Model^**#**^	
	***OR (***95%CI***)***	***P***	***OR*** (95%CI***)***	***P***
**Age (years)**				
18–25	*Ref*	–	*Ref*	**–**
26–29	0.51(0.28–0.93)	0.028	0.73(0.37–1.46)	0.371
30–34	0.56(0.30–1.02)	0.058	1.12(0.44–2.90)	0.808
≥ 35	1.51(0.90–2.54)	0.115	2.14(0.85–5.39)	0.106
**Ethnicity**				
Han	*Ref*	–	*Ref*	**–**
Non-Han	2.05(1.24–3.40)	0.005	2.24(1.28–3.93)	**0.005**
**Marital status**				
Not married	*Ref*	–	*Ref*	**–**
Married	0.71(0.46–1.08)	0.109	0.58(0.27–1.25)	0.164
Divorced or widowed	2.56(1.46–4.49)	0.001	1.46(0.58–3.64)	0.423
**Education level**				
Illiterate or elementary school	*Ref*	–	*Ref*	**–**
Junior high school	1.06(0.68–1.65)	0.789	1.51(0.94–2.45)	0.090
High school and above	1.67(0.86–3.25)	0.131	2.52(1.19–5.33)	0.016
**Monthly income (USD)**				
< $500	*Ref*	–	*Ref*	**–**
$500–$1000	0.49(0.33–0.74)	0.001	0.47(0.29–0.74)	**0.001**
> $1000	0.48(0.27–0.84)	0.010	0.29(0.16–0.55)	**0.001**
**Household registration**				
Local city	*Ref*	–	*Ref*	**–**
Other cities in this province	0.74(0.39–1.39)	0.348	0.77(0.39–1.50)	0.478
Other provinces	0.30(0.17–0.53)	< 0.001	0.30(0.16–0.55)	**0.001**
**Length of time working in current location**				
1 year and above	*Ref*	–	*Ref*	**–**
6–12 months	0.42(0.25–0.70)	0.001	0.47(0.27–0.81)	**0.006**
< 6 months	0.10(0.03–0.32)	< 0.001	0.14(0.04–0.45)	**0.001**
**Workplace**				
Middle tier	*Ref*	–	*Ref*	**–**
Low tier	4.06(1.47 ~ 11.18)	0.007	3.53(1.23 ~ 10.14)	**0.019**
**Average number of clients in the past month (continue variable)**				
	1.04(1.01 ~ 1.07)	0.041	1.03(1.01 ~ 1.05)	**0.043**
**Consistent condom uses with clients in the past month**				
No	*Ref*		*Ref*	
Yes	1.41(0.93 ~ 2.13)	0.101	1.79(1.12 ~ 2.86)	**0.016**
**Providing anal sex for clients**				
No	*Ref*	–	*Ref*	**–**
Yes	3.50(2.38 ~ 5.16)	< 0.001	2.36(1.54 ~ 3.60)	**< 0.001**
**Regular partner (boyfriend or husband)**				
No	*Ref*	–	*Ref*	**–**
Yes	1.86(1.12 ~ 3.09)	0.017	3.12(1.72 ~ 5.68)	**0.002**
**STD symptoms in the past 12 months**				
No	*Ref*	–	*Ref*	**–**
Yes	5.44(3.66 ~ 8.09)	< 0.001	4.09(2.62 ~ 6.40)	**< 0.001**
**HIV testing in the past 12 months**				
No	*Ref*	–	*Ref*	**–**
Yes	5.32(3.57 ~ 7.92)	< 0.001	5.16(3.21 ~ 8.30)	**< 0.001**
**Syphilis testing in the past 12 months**				
No	*Ref*	–	*Ref*	**–**
Yes	6.82(4.5 ~ 10.33)	< 0.001	6.90(4.21 ~ 11.22)	**< 0.001**
**Smoke in the past 12 months**				
No	*Ref*	–	*Ref*	**–**
Yes	0.57(0.33–1.01)	0.051	0.59(0.33–1.06)	0.076
**Sexual abuse in the past 12 months**				
No	*Ref*	–	*Ref*	**–**
Yes	3.60(2.11–6.15)	< 0.001	4.42(2.64–7.39)	**< 0.001**

Note: # Age, ethnicity, marital status, education level, monthly income, household registration and length of time working in the current location were adjusted for each other, all other variables were adjusted for age, ethnicity, marital status, education level, monthly income, household registration and length of time working in the current location

### Factors correlated with gonorrhea testing

In the multivariable analysis, gonorrhea testing was positively correlated with number of clients in the past month (a*OR* = 1.02, 95% CI:1.00–1.04), providing anal sex for clients (a*OR* = 1.82; 95% CI: 1.11–2.96), having regular partners (a*OR* = 2.59, 95% CI: 1.35–4.99), having STD symptoms over the past 12 months (a*OR* = 3.33, 95% CI: 2.03–5.46), HIV testing in the past 12 months (a*OR* = 3.94, 95% CI: 2.34–6.65), syphilis testing in the past 12 months (a*OR* = 3.27, 95% CI: 1.96–5.46) and sexual abuse in the past 12 months (a*OR* = 2.01, 95% CI: 1.08–3.72) after adjusting for age, ethnicity, marital status, the highest education level, marital status, annual income, household registration, and length of time working in the current location (Table 3).

**Table 3:** Factors associated with gonorrhea testing among FSW in Southern China, 2018 (*N* = 1207)

Characteristics	Crude Model		Adjusted Model^**#**^	
	***OR (***95%CI***)***	***P***	***OR (***95%CI***)***	***P***
**Age (years)**				
18–25	*Ref*	–	*Ref*	**–**
26–29	0.35(0.17–0.72)	0.005	0.45(0.19–1.05)	0.064
30–34	0.48(0.24–0.97)	0.041	0.81(0.26–2.53)	0.721
≥ 35	1.47(0.83–2.61)	0.185	1.70(0.56–5.10)	0.347
**Ethnicity**				
Han	*Ref*	–	*Ref*	**–**
Non-Han	2.39(1.37–4.15)	0.002	2.09(1.15–3.81)	**0.016**
**Marital status**				
Not married	*Ref*	–	*Ref*	**–**
Married	0.76(0.46–1.25)	0.284	0.65(0.25–1.69)	0.379
Divorced or widowed	2.74(1.45–5.19)	0.002	1.53(0.51–4.60)	0.450
**Education level**				
Illiterate or elementary school	*Ref*	–	*Ref*	**–**
Junior high school	0.81(0.50–1.30)	0.382	1.15(0.69–1.92)	0.586
High school and above	0.80(0.34–1.90)	0.613	1.20(0.47–3.06)	0.699
**Monthly income (USD)**				
< $500	*Ref*	–	*Ref*	**–**
$500–$1000	0.51(0.32–0.83)	0.006	0.57(0.34–0.96)	**0.033**
> $1000	0.56(0.29–1.05)	0.070	0.47(0.24–0.93)	**0.031**
**Household registration**				
Local city	*Ref*	–	*Ref*	**–**
Other cities in this province	0.69(0.31–1.54)	0.367	0.69(0.30–1.60)	0.384
Other provinces	0.48(0.24–0.96)	0.037	0.49(0.24–1.02)	0.056
**Length of time working in current location**				
1 year and above	*Ref*	–	*Ref*	**–**
6–12 months	0.51(0.29–0.91)	0.022	0.60(0.33–1.10)	0.098
< 6 months	0.10(0.02–0.41)	0.001	0.13(0.03–0.56)	**0.006**
**Workplace**				
Middle-level venue	*Ref*	–	*Ref*	–
Low-level venue	2.78(1.01 ~ 7.70)	0.049	1.95(0.68 ~ 5.59)	0.216
**Average number of clients in the past month (continue variable)**				
	1.02(1.00 ~ 1.04)	0.043	1.02(1.00 ~ 1.04)	**0.045**
**Consistent condom uses with clients in the past month**				
No	*Ref*	–	*Ref*	–
Yes	1.01(0.64 ~ 1.59)	0.969	1.15(0.69 ~ 1.92)	0.583
**Providing anal sex for clients**				
No	*Ref*	–	*Ref*	–
Yes	2.44(1.55 ~ 3.85)	0.001	1.82(1.11 ~ 2.96)	**0.017**
**Regular partner (boyfriend or husband)**				
No	*Ref*	–	*Ref*	–
Yes	1.50(0.86 ~ 2.62)	0.155	2.59(1.35 ~ 4.99)	**0.004**
**STD symptoms in the past 12 months**				
No	*Ref*	–	*Ref*	–
Yes	4.03(2.58 ~ 6.29)	< 0.001	3.33(2.03 ~ 5.46)	**< 0.001**
**HIV testing in the past 12 months**				
No	*Ref*	–	*Ref*	–
Yes	5.32(3.57 ~ 7.92)	< 0.001	3.94(2.34 ~ 6.65)	**< 0.001**
**Syphilis testing in the past 12 months**				
No	*Ref*	–	*Ref*	–
Yes	6.82(4.5 ~ 10.33)	< 0.001	3.27(1.96 ~ 5.46)	**< 0.001**
**Smoke in the past 12 months**				
No	*Ref*	–	*Ref*	–
Yes	0.87(0.49–1.55)	0.646	0.91(0.50–1.66)	0.761
**Sexual abuse in the past 12 months**				
No	*Ref*	–	*Ref*	–
Yes	8.64(5.53–13.49)	< 0.001	2.01(1.08–3.72)	**0.028**

Note: # Age, ethnicity, marital status, education level, monthly income, household registration and length of time working in the current location were adjusted for each other, all other variables were adjusted for age, ethnicity, marital status, education level, monthly income, household registration and length of time working in the current location

## Discussion

Although testing is an effective strategy to prevent the transmission of chlamydia and gonorrhea, our findings suggest that chlamydia and gonorrhea testing rates remain low among Chinese FSW. Only one in ten FSW in our study reported that they had tested for chlamydia and gonorrhea in the past year. Our study extends the existing literature by recruiting a large number of FSW on middle and low-level venues, and examining chlamydia and gonorrhea testing uptake and associated factors in China. Findings from this study provide an evidence for the future interventions on promoting chlamydia and gonorrhea testing uptake among FSW.

We found notably low levels of testing uptake of chlamydia and gonorrhea testing among Chinese FSW. In our study, chlamydia and gonorrhea testing rates were much lower than those reported in Germany (77.1 and 65.4%) [12] and previously reported among Chinese men who have sex with men (MSM) (28.5 and 30.6%) [2]. Additionally, FSW in this study reported high levels of inconsistent condom use (34.6%), which was similar to that in a systematic review among FSW in China [20], exposing them to the risk of contracting or transmitting HIV and frequent STD [21]. The low rates of testing uptake alongside highly risky sexual behaviors among FSW highlight the importance of promoting frequent testing among FSW in China. Chlamydia and gonorrhea screening guidelines have been released for sexually active women to promote the testing uptake in many countries which do not include China, such as United states [22], Australia [23] and England [24]. It has been proven that screening is an effective strategy for chlamydia and gonorrhea control [25]. There is an urgent need to explore effective strategy to improve the testing uptake of both gonorrhea and chlamydia in China.

We found that either HIV or syphilis testing was positively correlated with chlamydia and gonorrhea testing in this study. This finding was in line with studies conducted among MSM [2, 26]. This might be partly due to the comprehensive HIV and syphilis testing system in China, which might act as a gateway for FSW to improve awareness of other STD, such as chlamydia and gonorrhea [14]. Meanwhile, the FSW who had HIV testing tended to have higher awareness of STD testing [14]. Although there are many possible ways for promoting chlamydia and gonorrhea testing, such as home-based testing or self-collection [15], outreach services [27] and pay-it-forward [28], the effect is still not ideal. There are many proven effective strategies around the world in response to the low HIV and syphilis testing, such as voluntary counseling and testing (VCT) [29], provider-initiated HIV/syphilis testing and counseling (PITC) [30]. Given the correlation existing between chlamydia/gonorrhea testing and HIV/syphilis testing, integrating chlamydia and gonorrhea testing into the well-established HIV or syphilis testing programs has the potential to increase test uptake among FSW. Besides, reducing stigma from health care providers [31–33] and increasing social support from intimate partners [34] can promote chlamydia and gonorrhea testing among FSW. Our data showed that most of FSW were immigrants (77.6%). Community-based STD prevention programs can help FSW establish linkages to local health care providers and reduce related stigma [35]. Although social support is positively correlated with condom use, intimate partners who are reluctant to use condoms may also contribute to the spread of STD [36]. Future STD prevention intervention efforts need to focus on the positive effect of social support from intimate partners for FSW and maximize the protective role of social support in STD risk reduction [37].

Our results showed that FSW who have higher risk of STD infection were more likely to have chlamydia and gonorrhea testing in this study, such as low-tier FSW [6] and FSW who had a greater number of clients. This finding was similar with results reported in previous studies about HIV testing among FSW and chlamydia/gonorrhea testing among MSM [38, 39]. This may be partly attributable to higher risk perception of chlamydia and gonorrhea infection among those FSW. Although those FSW were more likely to test for chlamydia and gonorrhea, the testing rates among them were still low (only 8%, 86/1075).

There were several limitations of our study. First, participants in this study were not randomly recruited, so they might not accurately represent Chinese FSW. Second, the vulnerability of the self-reported information to social desirability bias may result in misinterpretation in this study, particularly those related to sexual behaviors. Third, this study was conducted among FSW in cities with rich experience in STD prevention. The results of this study may not be generalizable to FSW in cities that are not experienced in STD prevention. Last, this survey cannot distinguish the condom use between their clients and their stable partners (husband or boyfriend).

## Conclusions

The testing uptake of chlamydia and gonorrhea in Chinese FSW is low, alongside with a high level of engagement in risky sexual behaviors, which indicates a high risk of chlamydia and gonorrhea infection. Chlamydia and gonorrhea testing could be integrated into HIV and syphilis testing promotion programs to achieve further public health impact. Intervention to improve the availability of chlamydia and gonorrhea testing services is paramount.

## Supplementary Information

**Additional file 1.** English Questionnaire.

## Data Availability

The dataset used in the study are available from the corresponding author. on reasonable request.

## Abbreviations

FSW: Female sex workers
HIV: Human immunodeficiency virus
STD: Sexually transmitted disease
LMIC: Low and middle-income countries
OR: Odds ratios
CI: Confidence intervals
aOR: Adjusted odds ratio
MSM: Men who have sex with men
VCT: Voluntary counseling and testing
PITC: Provider-initiated HIV/syphilis testing and counseling
