# Supplementary material for: Uptake and correlates of chlamydia and gonorrhea testing among female sex workers in Southern China: a cross-sectional study
Source: BMC Public Health. 2021 Jul 28;21:1477. doi: 10.1186/s12889-021-11526-w (PMC8320049; doi:10.1186/s12889-021-11526-w)
Supplement: Supplementary file 1 — Additional file 1. English Questionnaire. [file 12889_2021_11526_MOESM1_ESM.docx]

Health Survey among FSW in Southern China

Number：

A01 City___________________________

A02 Investigation date ________________

A03 Sample source:

①Sauna/Bath Center ②Nightclub ③Karaoke Hall/Sing and Dance Hall/Bar ④Guesthouse/hotel ⑤ Foot washing house/hair salon ⑥Roadside shop/small restaurant ⑦Street ⑧Other_________

B01Age：

B02 Nation：①Han ②Other

B03 Marital status： ①Unmarried ②Married ③Divorced or widowed

B04 Place of household registration ①this city ②other cities in this province ③other provinces

B05 Living status :①Living alone ②Living with spouse ③Living with family other than spouse ④Living with friends

B06 Monthly income: ①1000 yuan and below ②1001-3000 yuan ③3001-5000 yuan ④5001-8000 yuan ⑤8000 yuan and above

B07 Education level: ①Illiterate ②Elementary school ③Junior high school ④High school or technical secondary school ⑤College and above

B08 Your local working time this time: ①One year or more ②6~12 months ③1~6 months ④less than one month

B09 In the past year, which of the following health services have you received locally? (Multiple choice)

①Publicity and education of sexually transmitted diseases ②Venereal disease examination or treatment ③AIDS knowledge and education ④HIV antibody testing and consultation ⑤Condom promotion and distribution ⑥Gynecological examination or treatment ⑦Syphilis testing and consultation ⑧Others (please specify)_______ ⑨ None of the above

B10 Have you experienced the following symptoms in the past year? (Multiple choice)

①Abnormal vaginal secretions (increased, purulent/bloodous secretions, etc.) ②Ulcers, blisters, erosions in the genital area③Pain in the lower abdomen during non-menstrual period ④ Cauliflower-like and papillary growths on the vulva ⑤ Itching or discomfort in the vulva⑥Others (please specify)___________⑦Without any of the above symptoms

B11 If the above symptoms (symptoms in item B10) occur, what would you do first?

①Check online or consult with friends ②Buy medicine for treatment by yourself ③See a doctor in the hospital ④No treatment ⑤Other _________

B12 If you go to the hospital to see a doctor, first choose the type of medical institution?

①Public general hospital ②Public maternity insurance hospital or maternity hospital ③Public dermatology hospital④Private general hospital ⑤Private dermatology hospital ⑥Private maternity hospital ⑦Private clinic

C01 How many guests do you have per week on average?

C02 In the past month, how often did you use condoms when you had sex with clients?

①None ②Sometimes ③Always

C03 Under what circumstances would you consider not using condoms? ①Receive familiar or fixed guests ②Guests suggest not to use ③Guests look healthy ④Guests pay more

C04 Do you have a regular sexual partner (boyfriend or husband)?①Yes ②No

C05 In the past month, how often did you use condoms when having sex with a regular partner?

①None ②Sometimes ③Always

C06 Have you ever provided oral sex to guests？①Yes ②No

C07 Have you ever used condoms when providing oral sex to your guests? ①Yes ②No

C08 Have you ever provided anal sex for your guests? ①Yes ②No

C09 Have you ever used condoms when providing anal sex to your guests?①Yes ②No

C10 Have you experienced violence from others at work in the past year ①Yes ②No

| **a.** **Clients** | | | |
| --- | --- | --- | --- |
| Rape | □Yes，____times | □No | □Do not remember |
| Beat | □Yes，____times | □No | □Do not remember |
| Abuse | □Yes，____times | □No | □Do not remember |
| **b.** **Fixed partner (don’t fill in if there is no fixed partner)** | | | |
| Rape | □Yes，____times | □No | □Do not remember |
| Beat | □Yes，____times | □No | □Do not remember |
| Abuse | □Yes，____times | □No | □Do not remember |

D1 Have you tried any of the following items in the past year? (You can choose more than one item, select "None" to skip Part D)

□None □Cigarette □Liqueur

□Drug（Heroin, morphine, marijuana, methamphetamine, magus, ecstasy, K powder, LSD, cocaine, cough drops, etc.）

D2 How often do you use these items?

|  | None | every day | Every two or three days | Every week | Every two weeks | Every month | <6 months | ≥6 months |
| --- | --- | --- | --- | --- | --- | --- | --- | --- |
| Cigarette |  |  |  |  |  |  |  |  |
| Liqueur |  |  |  |  |  |  |  |  |
| Drug |  |  |  |  |  |  |  |  |

D3 In the past year, did you use the above items before sexual intercourse?

|  | every time | sometimes（＜50%） | often（≥50%） | None |
| --- | --- | --- | --- | --- |
| Cigarette |  |  |  |  |
| Liqueur |  |  |  |  |
| Drug |  |  |  |  |

E1 Have you been tested for HIV in the past 3 months？ □Yes，___times □No

E2 Have you been tested for HIV in the past 12 months？ □Yes，___times □No

E3 Have you taken HIV self-test in the past 12 months？ □Yes，___times □No

E4 Have you been tested for syphilis in the past 3 months？ □Yes，___times □No

E5 Have you been tested for syphilis in the past 12 months？ □Yes，___times □No

E6 Have you been tested for gonorrhea in the past 3 months？□Yes，___times □No

E7 Have you been tested for gonorrhea in the past 12 months？□Yes，___times □No

E8 Have you been tested for chlamydia in the past 3 months？□Yes，___times □No

E9 Have you been tested for chlamydia in the past 12 months？□Yes，___times □No

Investigator's signature：______________ Supervisor's signature：__________
